# Targeting extra-oral bitter taste receptors modulates gastrointestinal motility with effects on satiation

**DOI:** 10.1038/srep15985

**Published:** 2015-11-06

**Authors:** Bert Avau, Alessandra Rotondo, Theo Thijs, Christopher N. Andrews, Pieter Janssen, Jan Tack, Inge Depoortere

**Affiliations:** 1 Translational Research Center for Gastrointestinal Disorders, University of Leuven, Leuven, Belgium

## Abstract

Bitter taste receptors (TAS2Rs) are present in extra-oral tissues, including gut endocrine cells. This study explored the presence and mechanism of action of TAS2R agonists on gut smooth muscle *in vitro* and investigated functional effects of intra-gastric administration of TAS2R agonists on gastric motility and satiation. TAS2Rs and taste signalling elements were expressed in smooth muscle tissue along the mouse gut and in human gastric smooth muscle cells (hGSMC). Bitter tastants induced concentration and region-dependent contractility changes in mouse intestinal muscle strips. Contractions induced by denatonium benzoate (DB) in gastric fundus were mediated via increases in intracellular Ca^2+^ release and extracellular Ca^2+^-influx, partially masked by a hyperpolarizing K^+^-efflux. Intra-gastric administration of DB in mice induced a TAS2R-dependent delay in gastric emptying. In hGSMC, bitter compounds evoked Ca^2+^-rises and increased ERK-phosphorylation. Healthy volunteers showed an impaired fundic relaxation in response to nutrient infusion and a decreased nutrient volume tolerance and increased satiation during an oral nutrient challenge test after intra-gastric DB administration. These findings suggest a potential role for intestinal TAS2Rs as therapeutic targets to alter gastrointestinal motility and hence to interfere with hunger signalling.

Taste perception on the tongue is essential in judging the quality of ingested food^1^. Bitter taste, sensed by 25 subtypes of the taste receptor type 2 (TAS2R) family of GPCRs, is an aversive stimulus and considered to be a toxicity detector^2^. Receptor stimulation results in activation of the taste-specific G-protein gustducin, stimulating a signalling cascade leading to the release of calcium from intracellular stores and activation of the membrane cation channel transient receptor potential M5 (TRPM5). This causes cell depolarization and neurotransmitter release, activating sensory nerves that communicate with the brain^3^. Recently, taste receptors have been identified in extra-oral tissues, suggesting additional functions for these receptors besides taste perception^4^. In the gut, taste receptors on endocrine cells sense nutrients to control the release of gastrointestinal hormones, which modulate ingestive behavior^5^. More specifically, TAS2Rs have been demonstrated on enteroendocrine cell lines and affect the secretion of the anorexigenic peptides cholecystokinin (CCK) and glucagon like peptide (GLP-1), while gavage of bitter tastants induced CCK-dependent hindbrain activation^6^,^7^,^8^,^9^. In addition, intra-gastric administration of a bitter mixture resulted in the release of the hunger hormone ghrelin, partially involving α-gustducin. This was associated with a short-term increase in food intake, followed by a long-term decrease in food intake, correlating with a decrease in gastric emptying^10^. These results suggest a potential role for bitter tastants in appetite regulation. In the central nervous system, functional bitter taste receptors were found in multiple regions of the rat and human brain^11^,^12^,^13^. Studies in airway smooth muscle have demonstrated the relaxing potential of bitter agonists on human and mouse smooth muscle^14^. Furthermore, inhaled bitter tastants counteracted asthmatic bronchoconstriction in a mouse model suggesting that TAS2Rs may represent novel targets for treating asthma.

Using a translational approach, the present study aimed to investigate whether TAS2Rs are also present on intestinal smooth muscle cells and influence gut contractility. The contractile potency of several bitter agonists was compared in different regions of the gut and the mechanism of action was determined. Additionally, the effect of intra-gastric administration of bitter tastants on gastric emptying in mice was further characterized. A human gastric smooth muscle cell culture was used to translate the molecular events induced by bitter agonists from mice to humans, while in two studies in healthy volunteers; the effect of bitter agonists on gastric accommodation, satiation and nutrient volume tolerance was studied.

## Results

### TAS2Rs and their downstream signalling molecules are expressed in mouse gut smooth muscle

**Figure 1:**
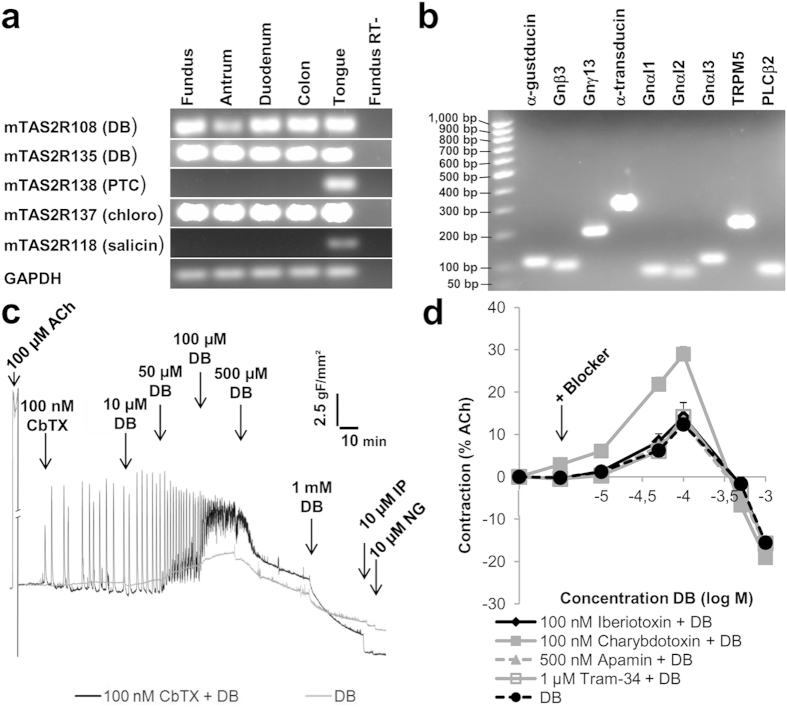
Expression of bitter taste signalling elements in mouse gut muscle and contractility responses to DB in the presence and absence of Ca^2+^-activated K^+^-channel blockers in mouse fundic smooth muscle strips. RT-PCR transcripts coding for (**a**) mTAS2Rs in mouse gut smooth muscle and tongue circumvallate papillae and (**b**) taste signalling molecules in mouse fundic smooth muscle tissue. (**c**) Representative tracing of the effects of increasing concentrations of DB, in absence or presence of 100 nM charybdotoxin (CbTX), on contractility in mouse fundic muscle strips, expressed as gram-force/mm^2^ (gF/mm^2^). (**d**) Concentration-response curves to DB in the absence (n = 30) or presence of CbTX (n = 71 mice), iberiotoxin (n = 4), TRAM-34 (n = 4) or apamin (n = 3) in mouse fundic strips. ACh = acetylcholine, IP = isoprotenerol, NG = nitroglycerine.

The expression of a selection of different bitter taste receptors and taste signalling elements was investigated in muscle strip preparations of mouse fundus, antrum, duodenum and colon via RT-PCR. No expression was shown for mTAS2R118 (salicin) and mTAS2R138 (phenylthiocarbamide (PTC)), while mRNA for mTAS2R108 (DB), mTAS2R135 (DB) and mTAS2R137 (chloroquine) was demonstrated throughout the gut with mouse tongue epithelium as positive control (Fig. 1a). Candidate TAS2R-coupled G-proteins such as α-gustducin and its corresponding β_3_γ_13_-subunits, α-transducin, Gαi1, Gαi2, Gαi3, were also expressed (Fig. 1b)^15^,^16^,^17^. The expression of phospholipase C β2 and TRPM5 confirmed the presence of the full canonical bitter taste signalling pathway.

### Bitter taste receptor agonists induce concentration-dependent changes in smooth muscle contractility

#### Effects of DB on smooth muscle contractility

In mouse fundic muscle strips, DB induced a concentration-dependent tonic contraction, which was maximal at 100 μM and amounted 12 ± 1% of the maximal response to ACh (P < 0.01) (Fig. 1c). At higher concentrations (1 mM), DB relaxed muscle strips strongly, up to 83 ± 17% of the maximal relaxation attained by nitroglycerine (NG) (P < 0.001).

To study the involvement of membrane K^+^ channels in the effect of DB on muscle contractility, fundic muscle strips were pretreated with blockers of different types of K^+^ channels. Blockade of large conductance Ca^2+^-activated K^+^ (BK_Ca_) channels by iberiotoxin (IbTx, 100 nM), intermediate conductance Ca^2+^-activated K^+^ (IK_Ca_) channels by TRAM-34 (1 μM) and small conductance Ca^2+^-activated K^+^ (SK_Ca_) channels by apamin (500 nM) did not affect the contractile response to DB (Fig. 1d). In contrast, pretreatment with the promiscuous K^+^ channel blocker charybdotoxin (CbTX, 100 nM) resulted, surprisingly, in an increased tonic contraction in response to DB (28 ± 2% of response to ACh) (P < 0.001), without affecting the relaxation at higher concentrations (Fig. 1c,d). Spikes of phasic contractions in fundic muscle strips (Fig. 1c) are a characteristic of CbTX-treatment and do not involve DB treatment.

**Figure 2:**
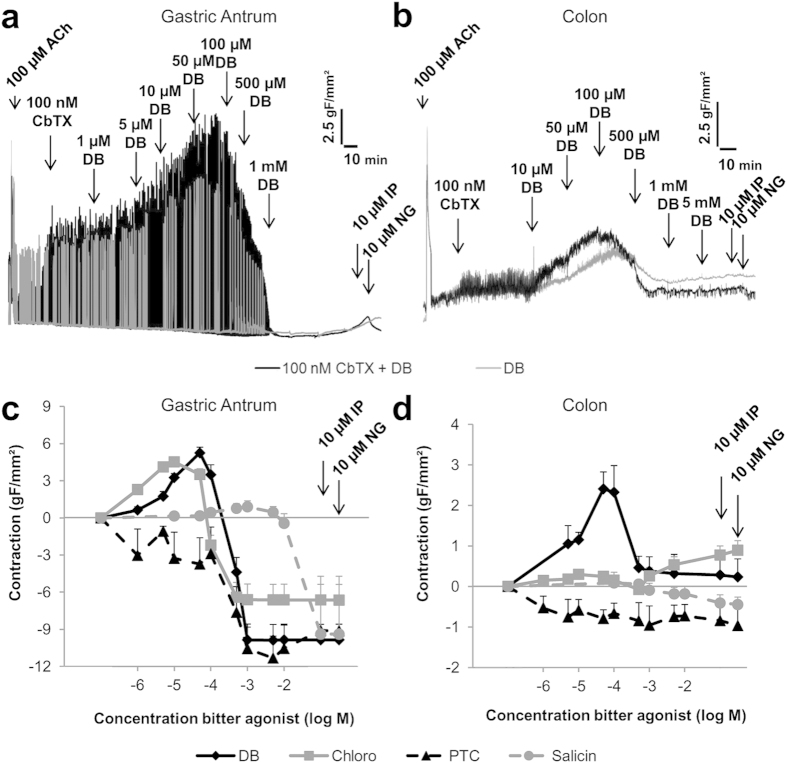
Specific contractility responses of various bitter taste receptor agonists in smooth muscle strips from different regions of the mouse gut. Representative tracing of the effects of increasing concentrations of DB, in the absence or presence of 100 nM CbTX, on contractility in mouse antral (**a**) and colonic (**b**) muscle strips. Cumulative concentration-dependent contractility changes induced by the bitter agonists DB, chloroquine, PTC and salicin, in the presence of 100 nM CbTX in mouse antral (**c**) and colonic (**d**) muscle strips. (N = 3–21 strips, n = 3–12 mice).

In the antrum (Fig. 2a) and the colon (Fig. 2b), a similar contractile pattern was observed with DB as in the fundus. In the antrum, DB reached a maximal increase in phasic activity, characteristic for antral smooth muscle, of 37 ± 7% (P < 0.001) and a maximal tonic contraction of 19 ± 3% (P < 0.001) in the colon, both at 50 μM. Pretreatment with CbTX resulted in a 2-fold and 1.4-fold increase in contractility in the antrum and colon respectively. At higher concentrations, DB inhibited the phasic activity in the antrum (P < 0.01) and the tonic contraction in the colon.

#### Effects of other bitter tastants on smooth muscle contractility

Chloroquine induced a significant increase (34 ± 3%, P < 0.001) in phasic activity of the antrum, followed by an inhibition at higher concentrations (P < 0.001) (Fig. 2c). No significant tonic contractions were observed in fundus or colon, but chloroquine relaxed (90% relative to NG, P < 0.001) fundic muscle at 500 μM (P < 0.001) (Fig. S1 and 2d). Phenylthiocarbamide did not induce a contraction in any of the tissues tested but relaxed fundic muscle strips, up to 51 ± 10% at 5 mM (P < 0.05 Supplementary Fig. S1) and completely inhibited phasic activity in the antrum (Fig. 2c, P < 0.001). PTC had no effect in the colon (Fig. 2d). Salicin did not influence contractility in any of the tissues tested (Fig. S1, 2c,d).

### Contractions in response to bitter tastants are mediated via TAS2R activation and the canonical taste signalling pathway

The DB-induced contraction was further characterized in fundic muscle strips and was shown to be muscle-specific, since blockade of neurotransmission with tetrodotoxin (3 μM) did not influence the contraction (Table 1).

**Figure 3:**
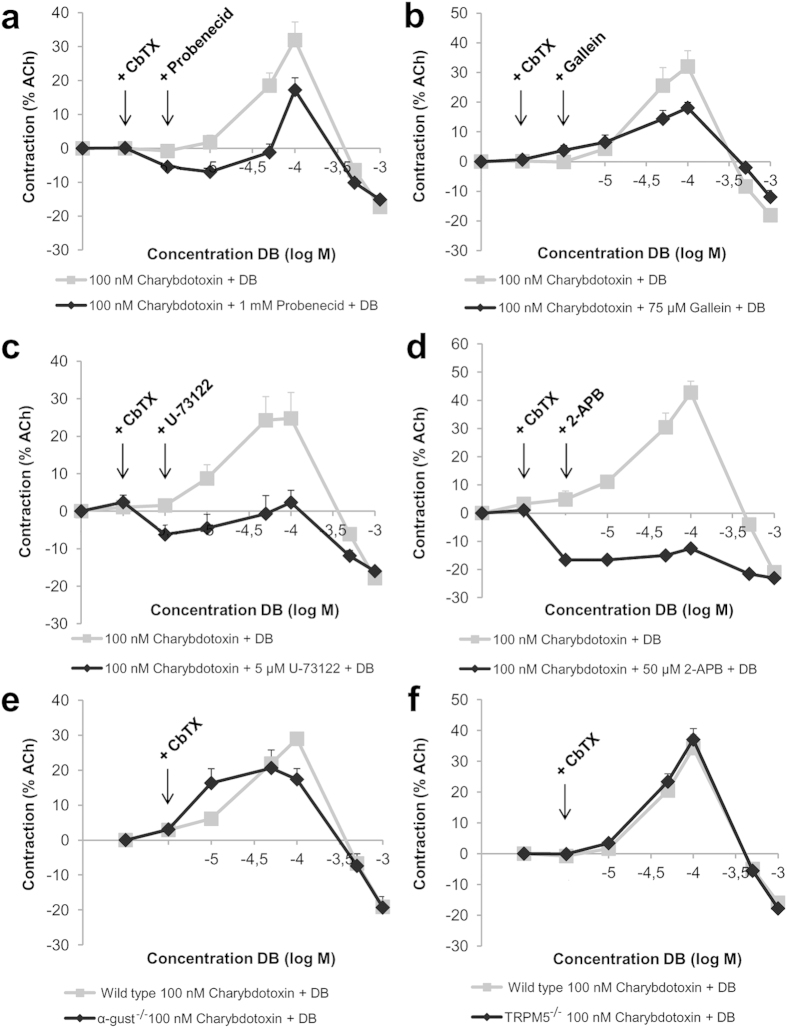
Effects of pharmacological blockers on DB-induced contractile responses in mouse fundic muscle strips and comparison of the responses to DB in fundic muscle strips of WT and α-gust^−/−^ or TRPM5^−/−^ mice. Effect of pharmacological blockers on contractility changes induced by DB in mouse fundic smooth muscle strips. CbTX-treated strips were pre-incubated with (**a**) probenecid (n = 7, P < 0.05), (**b**) gallein (n = 6, P < 0.001), (**c**) U-73122 (n = 6, P < 0.05) or (**d**) 2-APB (n = 4, P < 0.01) before addition of DB at increasing concentrations. Comparison of DB-induced contractions in WT (N = 15–26 strips, n = 5–21 mice) and (**e**) α-gust^−/−^ (N = 16 strips, n = 9 mice, P < 0.05) or (**f**) TRPM5^−/−^ (N = 12 strips, n = 3 mice, P > 0.05) mice in the presence of CbTX.

Involvement of the taste-signalling cascade was investigated with different blockers of the taste signalling pathway. Blockade of TAS2Rs with a TAS2R antagonist (probenecid, 1 mM; P < 0.05 vs vehicle-treated muscle strips) (Fig. 3a), the βγ-subunit of G-proteins (gallein, 75 μM; P < 0.001) (Fig. 3b), PLCβ (U-73122, 5 μM; P < 0.05) (Fig. 3c), the IP_3_-receptor (2-APB, 50 μM; P < 0.01) (Fig. 3d) or endoplasmic reticulum-depletion (thapsigargin, 10 μM; P < 0.001) (Table 1) all inhibited the contractions at lower concentrations, without influencing the relaxation at higher concentrations. Ryanodine (10 μM) on the other hand did not influence contractility (Table 1). In addition, contractions in strips from α-gustducin^−/−^ mice (α-gust^−/−^) were significantly lower compared to WT mice (P < 0.001) further confirming the involvement of taste signalling in the DB-induced contractions (Fig. 3e).

Ca^2+^-free buffer or pre-treatment of the strips with the L-type Ca^2+^-channel blocker nifedipine abolished (P < 0.01) contractions in response to DB indicating that influx of extracellular Ca^2+^ is important. TRPM5 seems not to be involved, since contractions were not different between muscle strips from WT mice and TRPM5^−/−^ mice (Fig. 3f). The Ca^2+^-influx appears to be dependent on activation of Ca^2+^-activated Cl^−^-channels, as pre-treatment with niflumic acid (20 μM; P < 0.01) also inhibited DB-induced contractions (Table 1).

Further downstream, involvement of Ca^2+^-dependent protein kinase C subtypes and ERK-signalling was shown via blockade of PKC_Ca_ with GF109203X (3 μM; P < 0.05) and MEK (PD98059, 10 μM; P < 0.05), both inhibiting contractions in response to DB. The Rho-associated protein kinase (ROCK) blocker Y-27632 (10 μM; P < 0.01) also abolished DB-induced contractions (Table 1).

### Relaxations in response to higher concentrations of bitter tastants do not involve the canonical taste pathway, but are PKC-dependent.

None of the previously tested blockers influenced the relaxations at higher concentrations. Neither did inhibition of cAMP/cGMP signalling with SQ-22536 (cAMP, 100 μM) or ODQ (cGMP, 10 μM). In contrast, the general PKC-inhibitor calphostine C (1 μM; P < 0.001) inhibited both DB-induced contractions and relaxations.

### Bitter taste receptor agonists inhibit gastric emptying in mice

**Figure 4:**
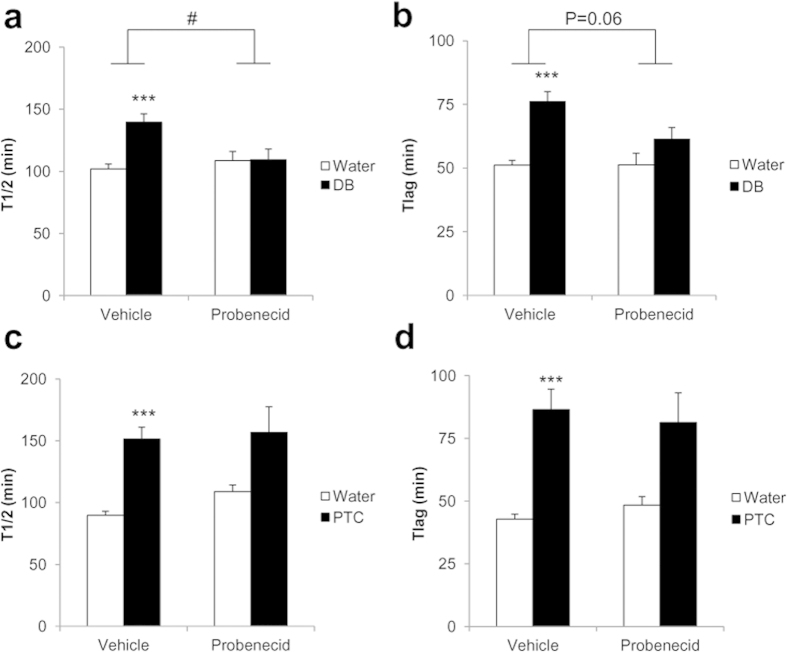
Effect of the bitter agonists DB or PTC on gastric emptying in mice in the absence or presence of the TAS2R antagonist probenecid. Effect of intra-gastric administration of bitter agonists on gastric half-excretion time (T_1/2_, **a**,**c**) and lag time (T_lag_, **b**,**d**). Mice (n = 12) were pretreated with vehicle or probenecid (i.p., 50 mg/kg), 15 min before oral gavage of DB (**a**,**b**) or PTC (**c**,**d**). Two-way ANOVA; *P < 0.05, ***P < 0.001 bitter vs water; #P < 0.05 probenecid vs vehicle.

Intra-gastric administration of DB and PTC significantly inhibited (P < 0.001) gastric emptying rate in mice as shown by an increase in gastric half-excretion time (T_1/2_) (DB: 38 ± 9%; PTC: 62 ± 8%) and lag time (T_lag_) (DB: 25 ± 4%; PTC: 44 ± 7%) (Fig. 4). Pretreatment of mice with probenecid (i.p., 50 mg/kg) inhibited the delay in gastric emptying induced by DB (treatment x blocker: T_1/2_: P < 0.05, T_lag_: P < 0.06), but not by PTC (Fig. 4).

### Bitter taste receptors and associated G-proteins are expressed in human gastric smooth muscle cells (hGSMCs)

**Figure 5:**
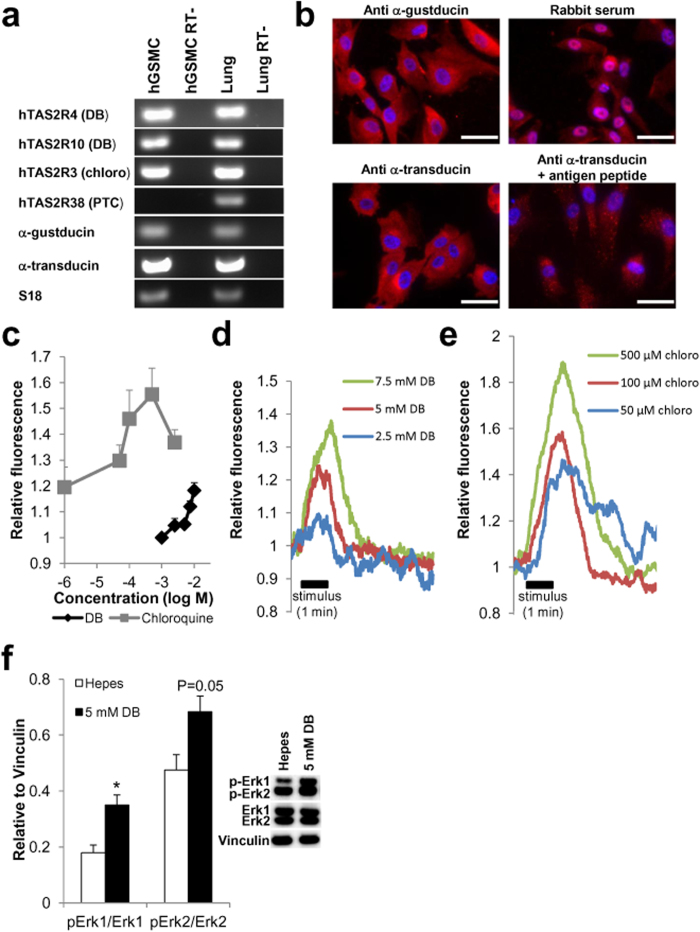
Expression of bitter taste signalling elements in human gastric smooth muscle cells and functional responses to bitter agonists in these cells. (**a**) RT-PCR transcripts coding for hTAS2Rs, α-gustducin and α-transducin in hGSMCs and human lung tissue. (**b**) Immunofluorescent stainings of hGSMCs for α-gustducin and α-transducin (scale bar = 50 μm). Negative controls include cells incubated with rabbit serum (α-gustducin) or with the specific antigen peptide (α-transducin). Nuclei are stained with DAPI. (**c**) Mean concentration-response curve of [Ca^2+^]_i_-rises in response to increasing concentrations of DB (n = 8 spots, P < 0.001) and chloroquine (n = 15 spots, P < 0.05) in hGSMCs. (**d**,**e**) Representative tracing of [Ca^2+^]_i_-rises in response to increasing concentrations of DB and chloroquine in a hGSMC. (**f**) Ratio (n = 4) of pErk over Erk, normalized to vinculin, in hGSMC in response to Hepes-buffer or 5 mM DB. *P < 0.05 vs Hepes buffer. Inlay: Representative western blot of Erk1/2 phosphorylation after stimulation.

In cultured hGSMCs mRNA expression for human bitter taste receptors hTAS2R3 (chloroquine), hTAS2R4 (DB), hTAS2R10 (DB) and their associated G-protein subunits (α-gustducin and α-transducin), but not hTAS2R38 (PTC) was demonstrated. Human bronchial tissue served as positive control (Fig. 5a). Protein expression of α-gustducin and α-transducin was confirmed by immunofluorescence (Fig. 5b).

### Bitter taste receptor agonists induce functional responses in human gastric smooth muscle cells

Both DB (Fig. 5d, P < 0.001) and chloroquine (Fig. 5e, P < 0.05) induced a concentration-dependent rise in [Ca^2+^]_i_ in hGSMCs loaded with the Ca^2+^-indicator Fluo-4 AM with chloroquine being more potent than DB (Fig. 5c).

Activation of the PKC-ERK signalling pathway was demonstrated by western blotting. Stimulation of hGSMC with 5 mM DB resulted in an increased phosphorylation of both Erk1 (P < 0.05) and Erk2 (P = 0.05) (Fig. 5f).

### DB inhibits gastric accommodation in healthy volunteers

**Figure 6:**
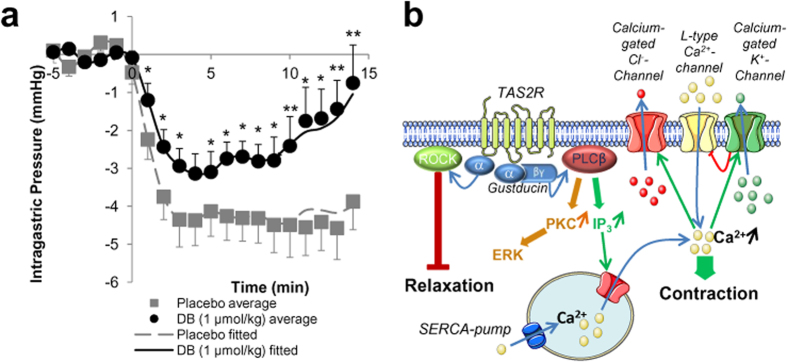
(**a**) Intra-gastric pressure in healthy volunteers (n = 12) during intra-gastric nutrient infusion after pretreatment with 1 μmol/kg DB or placebo. (*P < 0.05; **P < 0.01). (**b**) Overview of the proposed pathway for the contraction induced by DB in mouse fundic smooth muscle strips.

All volunteers (n = 12) completed the study without reporting adverse effects. Baseline intra-gastric pressure (IGP) prior to nutrient drink infusion was not different between placebo and DB. The course of the drop in IGP during nutrient infusion differed significantly (treatment: P = 0.01; treatment x time: P = 0.09) between placebo and DB treatment (Fig. 6a). IGP at nadir (placebo: −4.575 vs DB: −3.104 mmHg) tended (P = 0.06) to be more elevated during the DB condition but especially the return from the drop in IGP at nadir was faster after DB.

### DB increases satiation scores during liquid meal intake in healthy volunteers

After DB administration subjects (n = 13) felt satiated earlier (64 ± 6 min) compared with placebo treatment (74 ± 8 min; P = 0.01). Under vehicle conditions subjects ingested 998 ± 133 ml, while after DB treatment this was decreased to 869 ± 96 ml (P = 0.03). None of the other symptom ratings differed between DB and placebo (details not shown).

## Discussion

This study provided for the first time evidence that functional bitter taste receptors are present on smooth muscle cells of the human and mouse gut. Bitter tastants induced contractions *in vitro*, while intra-gastric administration of DB resulted in a TAS2R mediated delay in gastric emptying in mice. In humans, DB impaired gastric accommodation in response to intra-gastric nutrient infusion and decreased both time to reach full satiation and ingested nutrient volume in a nutrient drinking test. The presence of bitter taste receptors on both endocrine and smooth muscle cells in the gut suggests that bitter tastants might be used as novel stimuli to alter gastrointestinal motility and hence hunger signalling in man.

The contractile pattern induced by bitter tastants was region specific and agonist selective. DB induced a contraction in all tissues tested, followed by a relaxation at higher concentrations in fundus and antrum. Chloroquine followed largely the same pattern in the antrum, but was less potent in evoking contractions in fundus and colon. PTC only relaxed gut smooth muscle, while salicin had no effect on contractility. The discrepancy between these agonists might be a consequence of a differential bitter taste receptor expression in mouse gut smooth muscle. Both substances that to some extent cause contractions have a potential receptor present, but their ability to induce a response might differ depending on the sensitivity and number of TAS2Rs that are expressed. Alternatively, different TAS2Rs might elicit differential responses upon activation, explaining distinct responses for different bitter agonists. It needs to be emphasized that bitter compounds activate various TAS2Rs in different concentration ranges^18^. For instance in humans, DB can interact with 8 different TAS2Rs, chloroquine with 4 TAS2Rs of which two in common with DB and PTC does only interact with one receptor. In humans 80% of the 25 TAS2R subtypes have been de-orphaned, in mice only 6% of the 33 TAS2R subtypes^19^. The expression of a selection of mTAS2Rs was examined, based on homology to hTAS2Rs activated by the compounds used in the present study^20^.

Identifying actual TAS2Rs involved in the effects of bitter tastants on contractility is hampered by a lack of molecular tools. Reliable antibodies for TAS2Rs are unavailable and antagonists are scarce. TAS2R expression was confirmed by RT-PCR and their function studied using probenecid, the only commercially available molecule described to block a subset of TAS2Rs (namely hTAS2R16, −38 and −43 but not hTAS2R31)^21^. Probenecid reduced but did not abolish the DB-induced contractions in the mouse fundus suggesting that probenecid does not block all DB-activated TAS2Rs or acts with a different potency.

Experiments with muscle strips from α-gustducin^−/−^ mice indicate that the taste receptor-associated G-protein is involved in the observed effects of bitter tastants on gut contractility. However, since the contractions are not fully abolished, other G-proteins are probably in play as well. Indeed, TAS2Rs have been shown to couple to several G-proteins, including transducin and the inhibitory G-protein-subunits Gα_i_^15^,^16^,^17^, all expressed in gut smooth muscle. The taste receptor-associated cation channel TRPM5 seems, despite its expression in gut smooth muscle, not to be involved in the effects of bitter tastants on contractility.

The contraction induced by bitter tastants involves [Ca^2+^]_i_ increases and Ca^2+^-sensitization (summarized in Fig. 6b). The increase in [Ca^2+^]_i_ is mediated by the activation of the canonical taste signalling pathway and consequently depolarization of the cell, leading to further Ca^2+^-influx from the extracellular space. Rises in [Ca^2+^]_i_ in response to bitter tastants have been described in airway smooth muscle, but are being claimed to be part of the relaxation mechanism^14^ on the one hand, or have been considered too low to induce a contraction on the other hand^22^. A recent paper however described Ca^2+^ mobilization in the pulmonary artery in response to various bitter tastants, including DB, and a TAS2R-mediated vasoconstriction^23^. We are the first to describe a small contraction in response to bitter tastant-induced Ca^2+^-rises in mouse gut smooth muscle, enhanced by incubation with a promiscuous K^+^-channel blocker, charybdotoxin. This suggests that a K^+^-efflux, causing hyperpolarization, is being induced by bitter taste receptor agonists, inhibiting the contraction, possibly by inhibiting L-type Ca^2+^-channel activation^14^. The Ca^2+^-sensitizing effect of bitter tastants is mediated via activation of ROCK, known to lead to inhibition of myosin light chain phosphatase^24^.

Bitter tastants induced a relaxation or inhibition of contractile activity at higher concentrations in several regions of the gastrointestinal tract. The mechanism behind the relaxation induced by DB in the mouse fundus could not be fully elucidated, but appears to be TAS2R-independent. Involvement of BK Ca^2+^-activated K^+^-channels^14^ or a direct effect of the βγ-subunits of the G-protein on L-type Ca^2+^-channels, as described in airway smooth muscle^22^, could not be confirmed. However, a general PKC-inhibitor abolished the relaxations, possibly due to a direct attenuating effect of PKC on L-type Ca^2+^-channels, as reported for the inhibitory effects of bile acids on gut smooth muscle^25^.

Bitter tastants also induced functional effects *in vivo*. Intra-gastric administration of DB or PTC delayed gastric emptying in mice. Moreover, the effect of DB but not of PTC could be blocked by probenecid. This confirms that the effect of DB is TAS2R mediated, while that of PTC is not and is in agreement with the finding that mice are insensitive to the bitter taste of PTC^26^ and is further substantiated by the absence of mRNA expression for mTAS2R138 in the mouse gut. Some bitter agonists (chloroquine, salicin) are absorbed via the lumen and can gain access to the smooth muscle via the bloodstream^27^,^28^. Although benzoate is readily absorbed it is unclear whether this is also the case for denatonium benzoate^29^. Therefore, it needs to be emphasized that *in vivo* other modes of action for bitter agonists besides effects on smooth muscle cells are probably in play as well. For instance, DB receptors are expressed in the rat brain^11^ and also the release of gut peptides will play a part in the overall *in vivo* effect of bitter administration on gastric emptying^4^. A delay in gastric emptying correlating with a decrease in food intake in response to bitter tastants has previously been reported, and was shown to be independent of CCK or GLP-1 release^10^,^30^.

A primary cell culture of human gastric smooth muscle cells was used to determine whether the observed effects of bitter tastants in mouse smooth muscle strips were also representative for human smooth muscle. Bitter taste receptors, homologous to the ones identified in mouse tissue were found to be expressed, as well as α-gustducin and α-transducin. Stimulation of human gastric smooth muscle cells with bitter tastants evoked [Ca^2+^]_i_ rises and phosphorylation of ERK, and thus suggest the presence of a mechanism analogous to that described in mouse tissue.

Bitter agonists also induced functional effects in healthy volunteers, since intra-gastric administration of DB inhibited the drop in fundic tone in response to nutrient infusion. The functional relevance of this response was confirmed during a satiety drinking test, where intra-gastric administration of DB was associated with decreased nutrient volume tolerance and earlier satiation without inducing adverse symptoms. It still remains to be determined whether the effects described in our pre-clinical models are responsible for the observations in humans.

The actual physiological meaning of the dose-dependent changes in contractility in response to bitter tastants is not fully clear. Impaired accommodation, measured with the barostat or IGP monitoring, is associated with increased satiety and unexplained weight loss in functional dyspepsia^31^,^32^. Together with a possible inhibition of gastric emptying this may represent a system of highly orchestrated interactions that together with the aversive reactions elicited by bitter in the lingual system prevent toxic bitter compounds from being ingested and absorbed into the circulation. In the colon, an increased contractility, in combination with increased ionic secretions^33^ might be effective to expel pathogenic bacteria from the body. Interaction of bacterial quorum sensing molecules with TAS2Rs has already been shown in the airways^34^.

The current observations indicate potential therapeutic applications for bitter tastants in different disorders. The effect of bitter tastants on gastric accommodation or gastric emptying and hence on satiation or satiety might encompass a therapeutic potential in the treatment of obesity. The observed effects on contractility in the lower gut suggest possibilities in the treatment of ileus or constipation with bitter agonists. The regional specificity of a selective bitter agonist might make it possible to target specific regions. However, bitter tastants affect other tissues than mere the smooth muscle, such as the mucosal endocrine cells to affect hormone release^10^, and our observations *in vivo* are probably a summation of these effects. Long-term studies with bitter agonists in animals of disease might shed light on the therapeutic potential of TAS2Rs in the gut.

## Materials and Methods

### Animals

Wild type C57BL/6 (Janvier), α-gust^−/−^ (kindly provided by R. Margolskee, Monell Chemical Senses center) and TRPM5^−/−^ (kindly provided by K. Talavera, KU Leuven) mice were kept in the animal facility in 14–10 light-dark cycle with ad libitum access to chow and water. All experiments were approved by and conducted in accordance to the regulations of the ethical committee on animal experiments of the University of Leuven.

### Reverse transcription (RT) PCR

Total RNA was extracted from gut smooth muscle tissue and human gastric smooth muscle cells/lung tissue using the RNeasy fibrous tissue mini kit or RNeasy mini kit (Qiagen), respectively, and treated with the Turbo DNA-free kit (Ambion, Life technologies) to exclude genomic DNA contamination before reverse transcription using Superscript II Reverse Transcriptase (Invitrogen, Life technologies). 5 different samples per tissue were analyzed in triplicate, originating from 5 different mice. The RT- PCR reaction was performed as described previously^35^ and PCR-products were analyzed with agarose gel electrophoresis. The primers used are summarized in Supplementary Table S1. Amplicons for the TAS2Rs, α-gustducin and α-transducin in mouse fundic smooth muscle and hGSMC were extracted from the agarose gel with the Qiaquick gel extraction kit (Qiagen) and sequenced (LGC genomics) to confirm primer specificity. Amplicons of TAS2Rs that were not expressed in the target tissues were sequenced from the positive controls, mouse tongue and human lung.

### *In vitro* contractility studies in smooth muscle strips

Mucosal-free smooth muscle strips from fundus of WT, α-gust^−/−^ and TRPM5^−/−^ and antrum and colon of WT were suspended along their circular axis in a Krebs-filled tissue bath. Tension was measured isometrically in response to increasing concentrations (10 μM–10 mM) of the bitter agonists DB, chloroquine, salicin or PTC, in the presence and absence of different pharmacological blockers (described in supplementary materials) added 30 min before bitter stimulation^36^.

### ^13^C Octanoic acid breath test for gastric emptying

Gastric emptying was measured as previously described^35^. Mice (n = 12) were gavaged with either DB (60 μmol/kg) or PTC (30 μmol/kg)^10^ or water, as control, 30 minutes prior to start of the measurement. Probenecid (50 mg/kg) or vehicle was injected intraperitoneally, 15 min before gavage. Control experiments were run during the first 4 weeks until stable gastric emptying times were obtained. Mice were then assigned, using a cross-over design, to the following treatments: vehicle+water, vehicle+bitter, probenecid+water and probenecid+bitter. Between each treatment a wash-out period of one week was introduced.

### Culture and immunocytochemistry of human gastric smooth muscle cells (hGSMC)

hGSMCs were purchased from Innoprot and cultured according to the manufacturer’s guidelines. The cells were fixated and stained overnight at 4 °C with rabbit anti-α-gustducin (1:200, #79760, Abcam) or rabbit anti-α-transducin (1:500, SC-390, Santa Cruz Biotechnology). Negative controls included the addition of normal rabbit serum (1:200) instead of the primary antibody (α-gustducin) or blocking the primary antibody (1:500) with a 5x excess of its specific blocking peptide (α-transducin, SC-390 P, Santa Cruz), according to the manufacturer’s guidelines. Cells were stained with Alexa Fluor 594 donkey anti-rabbit IgG (1:750, Invitrogen, Life technologies) as secondary antibody and DAPI (1:1000, Invitrogen, Life Technologies) for 2 hours.

### Western Blotting

Total protein from hGSMCs was separated using SDS-PAGE and transferred on a PVDF membrane. Membranes were incubated overnight at 4 °C with primary antibodies: rabbit anti-phospho-p44/42 MAPK (1:1000, #9101, Cell signalling) and rabbit anti-p44/42 MAPK (1:1000, #9101, Cell signalling) or mouse anti-vinculin (1:10000, Sigma-Aldrich) as a protein-loading control. Secondary antibodies used were peroxidase conjugated goat anti-rabbit IgG or goat anti-mouse IgG (1:5000, Sigma-Aldrich) (1h, RT). Bands were quantified by relative densitometry and normalized to vinculin, using ImageQuant TL 8.1 (GE Healthcare).

### Calcium imaging

hGSMCs, loaded with 10 μM Fluo-4 AM, were stimulated via a local perfusion system (1 ml/min) with increasing concentrations of DB or chloroquine for 60 sec. Changes in [Ca^2+^]_i_ were reflected as change in Fluo-4 AM fluorescence intensity and recorded at 525/50 nm using an inverted Zeiss Axiovert 200M microscope (Carl Zeiss), with TILL Poly V light source (TILL Photonics) and a cooled CCD camera (PCO Sensicam QE). Regions of interest were drawn and average fluorescence signals were normalized to baseline, all using Igor Pro (Wavemetrics).

### Gastric accommodation and satiation drinking test in humans

Detailed protocols of these experiments can be found in supplementary materials. In short, in a first protocol, healthy volunteers (n = 12, 5 males, mean age: 30.6 ± 2.7; mean BMI: 23.8 ± 1.2) underwent two intra-gastric pressure (IGP) studies, at least one week apart, with administration of saline or DB in a single-blind fashion. After an overnight fast, a high-resolution solid-state manometer system (Manoscan 360, Sierra Scientific Instruments) and nutrient catheter were positioned through the nose in the stomach. Following a stabilization period of at least 15 minutes, either 1 μmol/kg DB or vehicle was infused through the nutrient catheter, followed by a 5 ml water flush. After another 30 minutes, nutrient drink (Nutridrink, Nutricia; 630 KJ) was infused (60 ml/min) directly in the stomach until maximum satiation. Epigastric sensations (fullness, nausea, bloating, belching, epigastric pain and abdominal cramps) were scored every 5 min using a 100 mm visual analogue scale.

In a second protocol, volunteers received an intra-gastric administration of saline or DB in an identical manner as in the first protocol. Thirty minutes after drug administration, subjects were requested to maintain intake of the liquid meal (Nutridrink, Nutricia) at a fixed rate until full satiation. All procedures were approved by and conducted in accordance to the guidelines of the Ethics Committee of the Leuven University Hospital.

### Statistical analysis

Results are presented as mean ± SEM. Muscle tension in muscle strips in response to bitter agonists (gF/mm^2^) was calculated as the median tension for each dose (fundus and colon) or the difference of the 99.5 and 2 percentile (antrum). The latter is done to reflect changes in the phasic activity, characteristic for antral muscle strips. The effects of different doses of bitter agonists on muscle tension (% of maximal contraction in response to ACh) were analyzed using one-way ANOVA, followed by planned comparisons (STATISTICA 12; Statsoft). Effects of pharmacological agents on basal muscle tension (% ACh) were analyzed using a paired student’s t-test. Effects of pharmacological agents on concentration-dependent contractions were analyzed with repeated measures ANOVA, comparing to representative, non-treated strips from the same animal. Effects of pharmacological agents on maximal relaxation were analyzed comparing maximal relaxation in response to treatment to the maximal relaxation in response to 10 μM NG with a paired student’s t-test. Differences in gastric emptying were analyzed with two-way ANOVA. Effects of bitter tastants on Ca^2+^-release and ERK-phosphorylation in hGSMCs were analyzed with one-way ANOVA and an unpaired student’s t-test respectively. Differences in intra-gastric pressures between placebo and DB treatment in healthy volunteers were determined using a repeated measures mixed model analysis (SAS), while effects on maximal satiation time, nutrient volume intake and epigastric symptoms were analyzed with a paired student’s t-test. Significance was accepted at the 5% level.

## Additional Information

**How to cite this article**: Avau, B. *et al.* Targeting extra-oral bitter taste receptors modulates gastrointestinal motility with effects on satiation. *Sci. Rep.* **5**, 15985; doi: 10.1038/srep15985 (2015).

## Supplementary Material

Supplementary Figure S1 and Supplementary Methods

## Figures and Tables

**Table 1:** Maximal contractions in response to DB (100 μM) in the presence of CbTX in mouse smooth muscle strips.

Blocker (Concentration)	Target of blocker	Effect of blocker (% ACh)	Change in maximal contraction in absence vs presence of blocker
Tetrodotoxin (3 μM)	Na^+^_v_-Channels	0 ± 0%, P > 0.05	32 ± 6% vs 26 ± 3%, P > 0.05 (n = 4)
Indomethacin (10 μM)	Prostaglandin synthesis	−3 ± 0%, P > 0.05	31 ± 5% vs 31 ± 8%, P > 0.05 (n = 5)
Nifedipine (1 μM)	L-type Ca^2+^-channels	−6 ± 3%, P > 0.05	24 ± 7% vs −3 ± 1%, P < 0.01 (n = 3)
Niflumic Acid (20 μM)	Cl^−^_Ca2+_-channels	−12 ± 2%, P < 0.01	50 ± 7% vs 24 ± 5%, P < 0.01 (n = 5)
Probenecid (1 mM)	TAS2R	−5 ± 1%, P > 0.05	33 ± 6% vs 23 ± 4%, P < 0.05 (n = 7)
Gallein (75 μM)	βγ-subunit of G-protein	4 ± 2%, P > 0.05	32 ± 5% vs 14 ± 2%, P < 0.001 (n = 6)
U-73122 (5 μM)	PLCβ	−6 ± 3%, P > 0.05	23 ± 8% vs 9 ± 2%, P < 0.05 (n = 6)
ODQ (10 μM)	cGMP	2 ± 4%, P > 0.05	31 ± 4% vs 31 ± 8%, P > 0.05 (n = 2)
SQ-22536 (100 μM)	cAMP	−6 ± 1%, P > 0.05	29 ± 9% vs 33 ± 5%, P > 0.05 (n = 5)
KT-5720 (1 μM)	PKA	−2 ± 1%, P > 0.05	39 ± 6% vs 29 ± 6%, P > 0.05 (n = 5)
Thapsigargin (10 μM)	SERCA-pump	34 ± 1%, P < 0.01	13 ± 5% vs −18 ± 9%, P < 0.001 (n = 5)
2-APB (50 μM)	IP_3_-receptor	−17 ± 2%, P < 0.05	34 ± 5% vs 4 ± 2%, P < 0.01 (n = 4)
Ryanodine (10 μM)	Ryanodine receptor	4 ± 2%, P > 0.05	39 ± 9% vs 21 ± 6%, P > 0.05 (n = 4)
Calphostin C (1 μM)	All PKC	−3 ± 4%, P > 0.05	49 ± 1% vs 4 ± 1%, P < 0.001 (n = 3)
GF109203X (3 μM)	Ca^2+^-dependent PKC	−5 ± 1%, P > 0.05	35 ± 6% vs 14 ± 4%, P < 0.05 (n = 4)
Y-27632 (10 μM)	ROCK	−15 ± 2%, P < 0.01	41 ± 4% vs 0 ± 0%, P < 0.01 (n = 3)
PD-98059 (10 μM)	MEK	−6 ± 2%, P > 0.05	23 ± 7% vs 12 ± 1%, P < 0.05 (n = 5)
SB-203580 (10 μM)	p38MAPK	−8 ± 1%, P > 0.05	27 ± 10% vs 32 ± 4%, P > 0.05 (n = 4)

The effects of different pharmacological blockers on basal muscle tension are represented as %ACh and are compared to the baseline tension using a paired student’s t-test. The influence of blockers on DB-induced contractions is compared between vehicle and blocker-treated strips using repeated measures ANOVA.
