# Supplementary material for: Targeting extra-oral bitter taste receptors modulates gastrointestinal motility with effects on satiation
Source: Sci Rep. 2015 Nov 6;5:15985. doi: 10.1038/srep15985 (PMC4635351; doi:10.1038/srep15985)
Supplement: Supplementary Figure S1 and Supplementary Methods [file srep15985-s1.doc]

**Supplementary information for:**

**Targeting extra-oral bitter taste receptors modulates gastrointestinal motility with effects on satiation.**

Bert Avau, Alessandra Rotondo, Theo Thijs, Christopher N. Andrews, Pieter Janssen, Jan Tack, Inge Depoortere

**Supplementary Figure S1:**


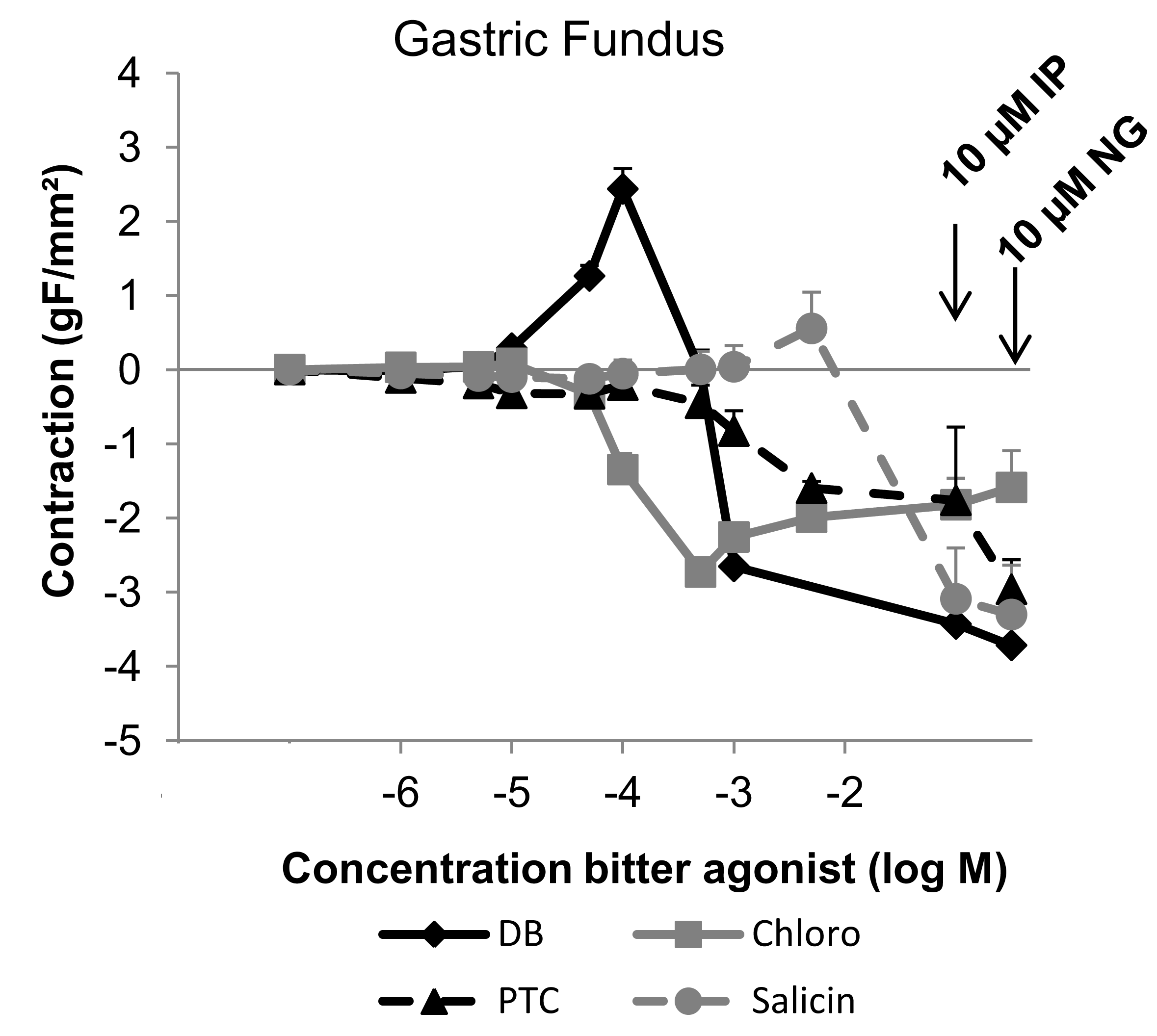


Cumulative concentration-dependent contractility changes induced by the bitter agonists DB, chloroquine, PTC and salicin, in the presence of 100 nM CbTX in mouse fundic muscle strips (n=4-71 mice).

**Supplementary methods**

**Chemicals used**

Apamin, chloroquine (chloro), denatonium benzoate (DB), GF109203X, indomethacin, niflumic acid, phenylthiocarbamide (PTC) and probenecid were purchased from Sigma Aldrich (St Louis, Missouri,USA).Salicin was purchased from Alfa Aesar (Karlsruhe, Germany). Charybdotoxin, iberiotoxin, ryanodine, tetrodotoxin, thapsigargin and TRAM-34 were purchased from Alomone labs (Jerusalem, Israel). PD-98059 and SB-203580 were purchased from Calbiochem, Merck (Darmstadt, Germany). 2-APB, calphostin C, gallein, KT-5720, nifedipine, ODQ, SQ-22536, U-73122 and Y-27632 were purchased from Tocris Bioscience (Ellisville, Missouri, USA).

Supplementary Table S1: Primer sequences used for RT-PCR

| Gene | Forward | Reverse | | Product size |
| --- | --- | --- | --- | --- |
| **Mouse primers** | | | | |
| GAPDH | CCCCAATgTgTCCgTCgTg | | gCCTgCTTCACCACCTTCT | 84 bp |
| mTAS2R108 (DB) | gATTTCAgCCCTCACCACTC | | AgTTCAggACCAAAgAggCTAC | 140 bp |
| mTAS2R135 (DB) | TgggCATgAAATggTTgCTC | | gAACAgAgCCAgATggTTgC | 211 bp |
| mTAS2R138 (PTC) | AAgCCATCCTCACTCTCTgg | | AgAAgCggACAATCTTggAg | 96 bp |
| mTAS2R137 (chloro) | gCgCACTgCTCTTATCCTgT | | ggCCTCAgCACTCTgATCTC | 278 bp |
| mTAS2R118 (salicin) | gCCTgTCCTTTTATTTTggAAg | | ggAAgATggggTgAgTgAAg | 128 bp |
| α-gustducin | CACCTCCATTgTTCTgTTTCTTAAC | | gCATCTTCAAATgTgTTTggTC | 114 bp |
| α-transducin | AATCgCATgCACgAgTCTTTg | | AgAgCCCACAgTCCTTgAgg | 340 bp |
| Gnβ3 | gAgTCAgACATCAATgCCATCT | | TCTgCCCTCAggTCAAAgAg | 98 bp |
| Gnγ13 | CCAgATgAAgAAggAggTAg | | gTCACACCTTACAgAgAgTg | 214 bp |
| Gnαi1 | ACgATTCggCAgCgTACT | | ATCCTgCTgAgTTgggATgT | 74 bp |
| Gnαi2 | TCAATgACTCAgCCgCTTAC | | gggATgTAgTCACTCTgTgCAA | 64 bp |
| Gnαi3 | gATTgATTTTggggAATCTgC | | AATCACgCCTgCTAgTTCTgA | 106 bp |
| PLCβ2 | CAgTggACCgCATTgATgT | | ACAggAACTgCCCAgAgATg | 69 bp |
| TRPM5 | gTCTggAATCACAggCCAAC | | gTTgATgTgCCCCAAAAACT | 234 bp |
| **Human primers** | | | | |
| S18 | ACCAACATCgATgggCggCg | TggTgATCACACgTTCCACCTCA | | 157 bp |
| hTAS2R4 (DB) | gAATCCCCAgACgAAgCTC | gTCCCCATATCCATCCCTgC | | 129 bp |
| hTAS2R10 (DB) | CCTTTggAgACACAACAggC | gACCCCAgggATAgATggCT | | 221 bp |
| hTAS2R38 (PTC) | AggCCCACATTAAAgCCCTC | CAgCTCTCCTCAACTTggCA | | 204 bp |
| hTAS2R3 (chloro) | TCCTCTggCTCAAgTggAgA | ATTTTgCAgCATCTgCCgTg | | 296 bp |
| α-gustducin | ATACCCTggAAgATggTggCATgA | TTCAgATgCCCTTTCAAAgCAggC | | 101 bp |
| α-transducin | ATCCgggCCATgACCACAC | TgAgggCTgCACAgAAAATgAT | | 430 bp |

**Gastric accommodation study protocol**

In a first protocol, healthy volunteers (n=12, 5 males, mean age: 30.6±2.7; mean BMI: 23.8±1.2) underwent measurement of intra-gastric pressure during nutrient challenge to assess the effect of DB on meal-induced gastric accommodation. The experimental procedure has been described in detail previously^1^. Volunteers underwent two IGP studies, at least one week apart, with administration of saline or DB in a single-blind fashion. After an overnight fast, a high-resolution solid-state manometer system (36 channels, 1 cm in between each channel, Manoscan 360, Sierra Scientific Instruments, Los Angeles, USA, Manoview analysis software v2.0.1) was positioned through the nose so that at least 1 sensor was positioned in the lower oesophageal sphincter (LES; detected as a clearly elevated pressure zone compared to oral and aboral areas), while IGP was measured as the average pressure of the first 5 pressure channels clearly below the LES or the pressure area influenced by the LES. A second catheter (Flocare, Nutricia, Bornem, Belgium) was positioned in the stomach through the nose or mouth that was used to infuse the bitter agonist and nutrient directly into the stomach. The tip of the infusion catheter was positioned approximately 5 cm below the LES and its position was verified by fluoroscopy.

After positioning the catheters, the subjects were positioned in a comfortable position with the knees bent (80 degrees) and the trunk upright, in a specially designed bed. Following a stabilization period of at least 15 minutes, either 1 µmol/kg DB or an identical volume of vehicle was infused through the nutrient catheter, followed by a 5 ml water flush. After another 30 minutes, nutrient drink (Nutridrink, Nutricia, Zoetermeer, the Netherlands; 630 KJ, 6 g proteins, 18.4 g carbohydrates and 5.8 g lipids per 100 ml) was infused directly in the stomach at a constant speed of 60 ml per min until maximum satiation. At 5-minute intervals, they scored other epigastric sensations (fullness, nausea, bloating, belching, epigastric pain and abdominal cramps) using a 100 mm visual analogue scale.

To avoid influence from movement artefacts and artefacts caused by coughing, sneezing, moving, or swallowing a moving median was calculated per channel from the original data. Per channel, a baseline value was calculated from the smoothed data as the average pressure in the 10 min immediately before the nutrient drink. The 5 pressure channels positioned below the LES were selected and averaged.

**Satiation drinking test protocol**

In a second protocol, we assessed the effect of DB on tolerance of orally ingested nutrients. Subjects (n=13, 5 males, mean age: 31.7±0.7; mean BMI: 22.6±0.3) underwent two nutrient challenge studies, at least one week apart, with intra-gastric administration of saline or DB in a double-blind fashion. An intra-gastric infusion catheter was positioned in the stomach through the nose or mouth as mentioned above. Following a stabilization period of 15 minutes, either 1 µmol/kg of DB or identical volume of vehicle was infused through the catheter, followed by a 5 ml water flush. Thirty minutes later, the infusion catheter was attached to a peristaltic pump (Minipuls2, Gilson, Villiers-Le-Bel, France) dispensing a liquid nutrient meal (Nutridrink, Nutricia, Belgium) in one of two beakers at a rate of 15 ml/min. Subjects were requested to maintain intake of the liquid meal at a rate equal to the dispensing rate, thereby alternating the beakers as they are filled and emptied. At 5-minute intervals, they scored their satiation and other epigastric sensations as described in the first protocol. Subjects were instructed to cease the meal intake when a score of 5 was reached^2^.

^1^ Janssen, P. et al., Intragastric pressure during food intake: a physiological and minimally invasive method to assess gastric accommodation. *Neurogastroenterol. Motil.* **23,** 316-322, e153-314 (2011).

^2^ Tack, J., Caenepeel, P., Piessevaux, H., Cuomo, R., and Janssens, J., Assessment of meal induced gastric accommodation by a satiety drinking test in health and in severe functional dyspepsia. *Gut* **52,** 1271-1277 (2003).
